# Animal Model Studies, Antibiotic Resistance and Toxin Gene Profile of NE Reproducing *Clostridium perfringens* Type A and Type G Strains Isolated from Commercial Poultry Farms in China

**DOI:** 10.3390/microorganisms11030622

**Published:** 2023-02-28

**Authors:** Mudassar Mohiuddin, Zhongfeng Song, Shenquan Liao, Nanshan Qi, Juan Li, Minna Lv, Xuhui Lin, Haiming Cai, Junjing Hu, Shaobing Liu, Jianfei Zhang, Youfang Gu, Mingfei Sun

**Affiliations:** 1 Key Laboratory of Livestock Disease Prevention of Guangdong Province, Key Laboratory of Avian Influenza and Other Major Poultry Diseases Prevention and Control, Ministry of Agriculture and Rural Affairs, Institute of Animal Health, Guangdong Academy of Agricultural Sciences, Guangzhou 510640, China; 2 Department of Microbiology, Faculty of Veterinary and Animal Sciences, The Islamia University of Bahawalpur, Bahawalpur 63100, Pakistan; 3 College of Animal Science and Technology, Anhui Science and Technology University, Chuzhou 233100, China

**Keywords:** necrotic enteritis, *C. perfringens*, *netB* gene, animal model studies, amoxicillin

## Abstract

Poultry necrotic enteritis (NE) is a complex and multifactorial disease caused by *Clostridium perfringens* types. Earlier, the disease was prevented and/or controlled through the addition of in-feed antibiotics and antimicrobial growth promoters (AGPs). The ban on the use of these agents as feed additives has been a major reason for re-emergence of this disease leading to huge economic losses to the world poultry industry. Understanding the pathogenesis of NE by developing an effective experimental model remains challenging and lacks consistency owing to the involvement of several critical factors involved in causing lesions of disease in the field. In this study, locally characterized *C. perfringens* types, i.e., ACP (toxinotype A), and GCP (toxinotype G), obtained from NE outbreaks on commercial farms in China (2020–2022), were used to experimentally induce NE in Specific-Pathogen-Free (SPF) chicks. The lesion scores observed on day 20 were 1.9 ± 1.10 (GCP strain) and 1.5 ± 1.08 (ACP strain), and both had significant difference as compared to the control group. The inclusion of fishmeal in addition to oral clostridial dose, i.e., fishmeal (day 7 onward) + Clostridia (7.5 × 10^8^ cfu/mL consecutively for 04 days) induced a lesion score of 2.0 ± 1.15 in respective groups. Use of coccidia (*Eimeria necatrix*) on day 9 followed by clostridia challenge enhanced the lesion scores to 2.5 ± 1.08 and 2.2 ± 1.23 for type G and type A strains, respectively. When both predisposing factors (coccidia + fish meal) were given together, i.e., fishmeal (day 7 onward) and coccidia (day 9) along with clostridia, the lesion scores were 3.2 ± 1.22 (GCP + coccidia + fish meal) and 3.0 ± 1.15 (ACP + coccidia + fish meal). These results were significantly different from group 1 (ACP) and 2 (GCP), in which only *C. perfringens* was used to induce NE. The clinical signs as well as histopathological lesions in experimentally induced groups were found similar as reported in the literature. The two type G strains identified in this study were also used for susceptibility testing against various drugs. Both strains were found to be resistant to amikacin, doxycycline, metronidazole, neomycin, nystatin, polymyxin B, streptomycin, and tetracycline. Variable susceptibility was seen against ceftriaxone, florfenicol, gentamicin, and kanamycin drugs. Amoxicillin, ampicillin, cefotaxime, ciprofloxacin, enrofloxacin, ofloxacin, and penicillin were effective drugs based upon their low level of resistance and therefore they might be preferred over other antimicrobial agents for proper treatment/prophylaxis of NE infections. Further studies are needed to study the pathogenesis of NE in detail in experimentally induced models along with continuous monitoring of the resistance pattern of *C. perfringens* strains in the field.

## 1. Introduction

Necrotic enteritis (NE) is a common enteric disease in poultry caused by *Clostridium perfringens* types [1]. Although the outbreaks of NE are sporadic, still the epidemiological data on the incidence of NE have reported it all around the globe and have categorized it as a major global threat to the poultry industry [2]. The condition occurs almost exclusively in broilers [3], although layers are also affected with outbreaks reported in 3–6 month old chickens [4]. During the last two decades, the prevalence of NE in layers has been increased not only in the Indian Subcontinent [5], but it has also been reported in USA and Europe [6,7]. The disease has re-emerged as a major problem for the poultry industry during the last decade, after the ban on the use of in-feed antimicrobials and antimicrobial growth promoters (AGPs) [8,9]. Although the primary causative agent is a commensal bacteria (*C. perfringens*), numerous additional predisposing factors have been identified that can lead to the more severe signs of this disease [10]. These contributing factors mainly include a protein-based diet, co-infection with other pathogenic microorganisms especially coccidia, and to a lesser extent other management- and stress-related factors [11,12,13].

The main causative agent, *C. perfringens,* is a spore forming, Gram positive, ubiquitous anaerobic bacteria found widely distributed in the environment [14]. The pathogenic behavior of this microorganism is dependent on the presence of virulence factors and enzymes, which together secrete more than 20 toxins [15]. The major toxins categorize it into seven toxinotypes including type A, type B, type C, type D, type E, type F, and type G [16]. This classification is based on the presence of *alpha*, *beta*, *epsilon*, *iota*, *enterotoxin,* and *netB* toxin genes. *Alpha* toxin gene alone in the absence of other major toxins is toxinotype A, *alpha* + *beta* + *epsilon* is toxinotype B, *alpha* + *beta* is toxinotype C, *alpha* + *epsilon* is toxinotype D, *alpha* + *iota* is toxinotype E, *alpha* + *enterotoxin* is toxinotype E, and *alpha* + *netB* is toxinotype G. More commonly, *C. perfringens* type G (*alpha* and *netB* gene) [17,18], and in some instances *C. perfringens* type A (*alpha* gene), has been involved in the pathogenesis of NE as reported in many naturally occurring disease investigations as well as animal model studies [19]. In our previous experimental trials, *netB*-positive isolate from a field outbreak of NE was able to induce severe disease even in the absence of predisposing factors [20]. Earlier *alpha* toxin has long been considered as critical factor in the pathogenesis of NE [19]; therefore, in this NE induction trial, *alpha*-positive type A isolate, refreshed using lab standardized protocol, was applied with and without predisposing factors. Successful induction of NE in chicken will add evidence to the argument that the virulence of NE strains includes factors other than *netB* also.

The clinical form of NE results in high mortality in flocks whereas the sub-clinical disease leads to reduced weight gain and poor feed conversion ratio [21]. It has also been found that *C. perfringens* strains isolated from NE outbreaks have shown resistance to routinely used antimicrobial agents, namely gentamicin and streptomycin, etc. [22,23]. In the present study, the distribution of different toxinotypes of *C. perfringens* at commercial large-scale farms in four different regions of China and their antimicrobial susceptibility against commonly used antibiotics has been studied. Theses local strains were identified and characterized through real-time PCR and were subsequently used to successfully induce NE in a disease induction model carried out with and without other predisposing factors.

## 2. Materials and Methods

### 2.1. Sample Collection

A total of 173 samples comprised of intestinal scrapings from dead birds having clinical signs of NE and cloacal swabs from NE suspected live birds were collected from chicken farms located in Anhui (*n* = 50), Guangzhou (*n* = 56), Guangxi (*n* = 48), and Fujian (*n* = 19) province, China. The intestinal scrapings from dead chickens were taken using sterile cotton swabs and kept in sealed plastic bags [24]. The samples were randomly collected from farms having outbreaks of NE located in South China and transported over ice to the lab for processing and stored under refrigerated conditions.

### 2.2. Sample Processing and Isolation of Bacteria

The fecal samples were processed following the protocol used by Mohiuddin et al. [25] and inoculated into freshly prepared cooked meat media (Guangdong Huankai Microbial Sci. and Tech. Co., Ltd., Guangzhou, China) at 37 °C for 24 h anaerobically in an anaerobic jar containing an anaerogen sachet (Oxoid, Basingstoke, UK). After incubation, a loopful from this broth culture was inoculated into Perfringens agar base containing D-cycloserine antibiotic supplement (Oxoid, Basingstoke, UK) and anaerobically incubated at 37 °C for 24 h.

### 2.3. Morphological Characteristics of C. perfringens

Black colonies on perfringens agar were picked aseptically and transferred on freshly prepared blood agar plates and subsequently on plates containing egg yolk agar (Guangdong Huankai Microbial Sci. and Tech. Co., Ltd., Guangzhou, China) to check hemolytic activity and presence of lecithinase enzyme in the isolated strains. Colonies having beta hemolysis forming double zone and the presence of lecithinase enzyme were characteristic and were stained with Gram’s stain to observe Gram-positive small to medium-sized rods containing sub-terminal spores. Pure colonies were preserved and used for further biochemical characterization and molecular analysis.

### 2.4. Biochemical Characterization of C. perfringens Strains

Pure single colony was inoculated into fluid thioglycolate (FT) broth (Guangdong Huankai Microbial Sci. and Tech. Co., Ltd., Guangzhou, China) and incubated at 37 °C for 15–18 h. Then, 50 uL of culture broth was drawn and used for biochemical testing as well as sugar fermentation reactions, namely, glucose, lactose, maltose, mannitol, sorbitol, sucrose, etc. The reaction conditions were anaerobic, and they were kept for 15–18 h at 37 °C after which results were recorded.

### 2.5. Identification of C. perfringens Toxinotypes through Quantitative Real-Time PCR (qPCR)

For toxinotyping, primers were used targeting CPA, CPB, ITX, ETX, CPE, TPEL, and NetB genes (Table 1). DNA extraction was carried out using pure colonies from 18 h culture of *C. perfringens* and centrifuged at 6000× *g* for 5 min. The pellet obtained was suspended in 200–250 µL of cold Tris EDTA (TE) buffer. DNA extraction was carried out following manufacturer’s instructions (Omega Bio-Tek, Norcross, GA, USA). The qPCR was carried out in a reaction mixture of 20 ul analyzed using thermocycler CFX96 Real-Time PCR Detection Systems (Bio-Rad Laboratories, Inc., Hercules, CA, USA). The mixture had concentration of different reagents as: TB green mix (Tli RNase H Plus, Takara) (10 µL), Primers (Forward 1 µL), (Reverse 1 µL), ddH20 (7 µL), and template DNA 1µL (150–200 ng). The qPCR reaction conditions were 95 °C for 30 s, followed by 34 cycles of denaturation at 95 °C for 15 s, annealing at 60 °C for 30 s, and dissociation steps 95 °C for 10 s, 65 °C for 5 s, and 95 °C for 5 s [26].

### 2.6. Confirmation of NetB Gene Sequence

For confirmation, primers were designed to amplify the CDS sequence of *netB* gene. The primer sequence was NetB F: 5′- TTGAAAAGATTAAAAATTAT-3′ and NetB R: 5′- GTAAGAAATCAAATCATATTG-3′. The *netB* gene was amplified using T100 thermocycler (Bio-Rad Laboratories, Inc., Hercules, CA, USA) in a reaction containing positive PCR product (1 µL), Premix Taq (LA Taq Version 2.0) 12.5 µL, primers (forward 0.5 µL), (reverse 0.5 µL), ddH2O (10.5 µL). The reaction conditions were 94 °C for 2 min, followed by 35 cycles of 98 °C for 10 sec, 47 °C for 30 s, and 68 °C for 1 min and final extension 72 °C for 10 min. The amplified product was electrophoresed on 2% agarose gel and observed under UV trans-illuminator. The purification of PCR product was performed through Gene Jet purification kit (OMEGA). After purification, Ligation was performed with T4 DNA Ligase, and recombinant plasmid (pMD18-T vector (Takara) containing *netB* gene sequence) was inserted into *E. coli* DH5α competent cells (Transgen Biotech, Beijing, China). Ligation was performed at 4 °C for overnight incubation. Transformation was confirmed by performing colony PCR of 4 single colonies by mixing with 10 µL ddH2O as a template. The cloned bacteria identified as positive by PCR were sent to Sangon Bioengineering (Shanghai) Co., Ltd. for sequencing.

### 2.7. Growth Curve for Clostridium Perfringens Type G Strains (D25) and (MZI)

The seed culture for each strain was inoculated into FT broth (Guangdong Huankai Microbial Sci. and Tech. Co., Ltd.) for 12 h at 37 °C anaerobically. Subsequently, each strain was re-inoculated into a new FT broth tube in 1:100 concentrations. The un-inoculated culture tube having FT media served as a blank (negative control). The OD_600 nm_ values were measured using a microplate reader after appropriate time intervals for both strains, and subsequently growth curves were plotted against time.

### 2.8. Animal Model Studies

#### 2.8.1. Chicks and Their Grouping

A total of 240 one-day old unvaccinated SPF chicks purchased from a commercial hatchery (Wen’s Foodstuffs Group Co., Ltd., Yunfu, China) were divided into 12 groups randomly with two replicates in each group and 10 chicks in each replicate.

#### 2.8.2. Bacterial and Coccidial Strains

*C. perfringens* type A (ACP), type G (GCP), and *Eimeria necatrix* strains isolated, identified and confirmed through sequencing were used for animal model studies. The strains were refreshed before use as mentioned in detail earlier [20] and used for inducing disease in SPF chicks.

#### 2.8.3. Experimental Design

Six groups namely: G1, G2, G3, G4, G5, and G6, of which G1 and G2 were *C. perfringens* (CP) infection groups. Chicks in these groups were given 7.5 × 10^8^ cfu/mL dose of either ACP or GCP once on days 14–17; G3 group was the *Eimeria necatrix* (EN) challenge group, and chicks were given oocysts (0.5 × 10^4^/mL); G4 and G5 were CP+EN group, 0.5 × 10^4^/mL EN sporulated oocysts once at day 9, and 7.5 × 10^8^ cfu/mL on day 14–17 of age with ACP and GCP strains, respectively; G6 was the control group in which chicks were given PBS (1 mL) at day 9, 14–17. All the chicks were humanely euthanized on day 20 to see the intestinal lesions. The chicks were fed a wheat-based diet (crude protein 18%) and had ad-libitum access to feed and water.

The rest six groups namely: G7, G8, G9, G10, G11, and G12, of which G7 and G8 were CP-infected groups, i.e., chicks were fed once at 14–17 days of age with ACP and GCP strain (7.5 × 10^8^ cfu/mL), respectively; G9 group was the Eimeria treatment group given sporulated oocysts once at the dose rate of 0.5 × 10^4^/mL on day 9; G10 and G11 were CP+EN groups (G10 was given ACP (7.5 × 10^8^ cfu/mL) day 14–17+ EN (0.5 × 10^4^ oocysts/mL) day 9) and (G11 was given GCP (7.5 × 10^8^ cfu/mL) day14–17+ EN (0.5 × 10^4^ oocysts/mL) day 9); the G12 group was the blank control group given PBS 1 mL on the respective days as in treatment groups. Five chicks from each group were slaughtered on day 20 to see the intestinal lesions. The chicks were fed a wheat-based diet (crude protein 18%) from day 1–7 and wheat-based high-protein diet (18% crude protein wheat-based diet mixed with fish meal having 68% crude protein mixed in the ratio of 1:1). The chicks had ad libitum access to feed and water during the whole experiment (Table 2).

#### 2.8.4. Lesion Scoring and Histopathology

Lesion scoring was carefully performed by examining the small intestine from duodenum to ileum for each bird. The lesion scoring ranged from 0 to 6; no gross lesions or simply congestion of intestinal mucosa were marked as 0 and 1, with non-significant to be considered as NE. The scores 2 (1–5 foci), 3 (6–15 foci), 4 (≥16 foci), 5 (necrotic patches 2–3 cm), and 6 (typical diffuse necrosis) were noted as described by Keyburn et al. [27]. Biasedness was avoided by observing the intestines usually by two people at a time on all sampling days. All the segments of the small intestine were observed carefully for scoring, however the highest score for any segment in each bird was noted as final lesion score. In addition to duodenum, jejunum and ileum, caecum was also examined for coccidial lesion scoring. On day 20, duodenal, jejunal, and ileal segments in triplicate were randomly collected from each group. The tissues were fixed in 10% phosphate-buffered-formalin. The samples were sent for histopathology to observe the pathological changes in each segment of small intestine.

### 2.9. Antibiotic Susceptibility Testing

Antimicrobial susceptibility testing (AST) was performed by Kirby–Bauer disc diffusion method using guidelines given by the CLSI [28]. The antibiotics were chosen based on their use in poultry. The following antimicrobials (Oxoid) were evaluated: amoxicillin (10 µg), amikacin (30 µg), ampicillin (10 µg), cefotaxime (30 µg), ciprofloxacin (5µg), ceftriaxone (30 µg), cephalexin (30 µg), doxycycline (5µg), florfenicol (30 µg), gentamicin (30 μg), kanamycin (30µg), metronidazole (5μg), neomycin (10µg), nystatin (100 µg), ofloxacin (5µg), penicillin (10 µg), polymyxin B (300 U), streptomycin (10 µg), and tetracycline (30 µg).

A single colony was picked from 24 h fresh culture of *C. perfringens* grown on perfringens agar and dispensed in saline solution. After thorough mixing, turbidity of culture was matched with standard McFarland solution and adjusted to 0.5 by addition of normal saline if the broth was more turbid. Bacterial lawn was prepared by dipping the sterile cotton swab in bacterial inoculum and spreading on the Mueller–Hinton agar plates. Antibiotic discs were placed at appropriate distance from each other on the inoculated plate. The area around the discs either showed variable zones of inhibition or in some cases no zone after 24 h of incubation at 37 °C. Zone diameter around each antibiotic disc was measured in millimeters (mm) (Table 3).

### 2.10. Statistical Analysis

Using IBM SPSS Statistics 23 version (IBM, New York, USA), one-way ANOVA was performed on the NE lesions scored data, which were processed group wise. The results were reported as the mean ± SEM, and small letters indicate significance in comparison to the control group (*p* < 0.05). Fisher’s exact test was used to compare the differences of NE incidence levels between various groups (*p* < 0.05).

## 3. Results

### 3.1. Morphological and Biochemical Characteristics of C. perfringens Strains

Morphologically identified characteristic colonies of *C. perfringens* on blood agar, perfringens agar, and egg yolk agar were confirmed biochemically through sugar fermentation reactions, gelatin liquefaction, and nitrate reduction tests. Pure colonies of *C. perfringens* fermented glucose, sucrose, maltose, and lactose, liquefied gelatin, and reduced nitrate while mannitol was found non-fermentative as reported earlier [29].

### 3.2. Positivity Rate (%) of Clostridium perfringens

A total of 44 intestinal samples giving a positivity rate of 25.4% were found positive for *C. perfringens*. The isolation rate in Anhui province was 20%, in Fujian 63.1%, in Guangxi 22.9%, and in Guangzhou was found to be 19.6% (Table 4).

### 3.3. Molecular Typing through Quantitative Real-Time PCR (qPCR)

*C. perfringens* types were identified using quantitative real-time (qPCR) targeting *cpa*, *cpb*, *etx*, *iota*, *tpel*, *cpe,* and *netB* toxin genes. All 44 isolates were confirmed as *C. perfringens* on the basis of presence of *cpa* (*alpha gene*), which is characteristic to all *C. perfringens* strains/types. The Tm value was 77.5, which corresponds to the positive control. Two out of forty-four isolates were found positive for *netB* gene also. The isolates having only *alpha* gene (CPA) (42 in number) were type A while two isolates which were positive for both *alpha* (*cpa*) and *netB* gene comprised type G isolates. The overall prevalence rate for *C. perfringens* was found to be 25.4%.

### 3.4. Cloning and Sequencing of NetB Toxin Gene

**Figure 1:**
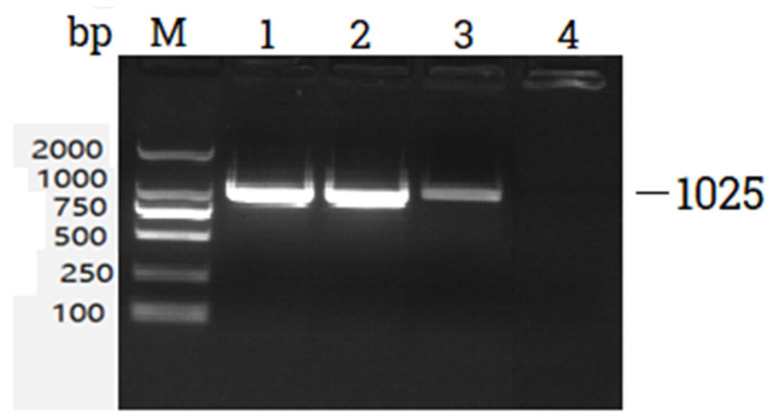
Agarose gel electrophoresis of PCR products. M: DNA marker (2000 bp), Lane 1: positive control, Lane 2,3: *netB* gene, Lane 4: negative control (no template DNA).

The PCR product positive for *netB* gene was used as a template for simple PCR using *netB* toxin gene primers. The amplified PCR product when run on 2% gel showed a band at 1025 bp (Figure 1). The band was cut from the gel and purified.

The amplified product recovered from the gel was ligated with pMD18-T easy vector. The ligated product was transformed into *E.coli* DH5α competent cells. The transformed colonies showed the positive single band, which was confirmed by sequencing (results not shown).

### 3.5. Growth Curve for Clostridium perfringens Type G Strains (D25) and (MZI)

**Figure 2:**
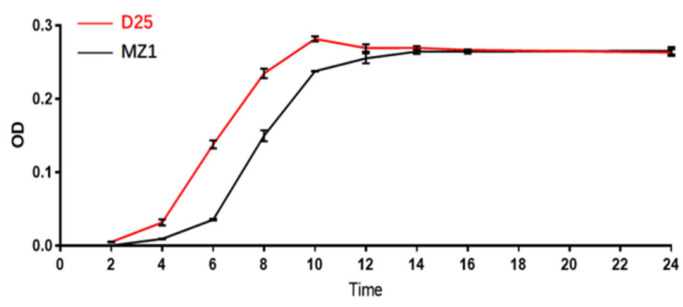
*C. perfringens* type G strains (D25 and MZI) growth curve.

The results have shown the adaptation period for D25 strain from 0–4 h; 04–10 h entered the logarithmic growth phase, and 10–24 h was stationary growth phase; for MZI strain, 0–6 h was adaptation period, 06–14 h logarithmic growth phase followed by 14–24 h stationary phase (Figure 2).

### 3.6. NE Lesion Score

The average NE lesion scores for G1 and G2 challenged with ACP and GCP strain were 1.5 ± 1.08 and 1.9 ± 1.10, respectively. A total of 5 out of 10 chicks infected with ACP strain (G1) developed NE lesions having score 2, while in case of GCP (G2), 7 out of 10 chicks developed mild NE (score 2). G3 group infected only with *Eimeria necatrix* at day 9 developed lesions which were not obvious for enteritis. Only two chicks had shown lesions in G3. When ACP and GCP strains were given along with coccidia strain as in G4 and G5; the G4 group developed NE lesions in 8 out of 10 chicks (6 chicks having score 3, while 2 had score 2); the G5 group developed NE lesions in 9 out of 10 chicks (1 chick: score 4, 4 chicks: score 3, 4 chicks: score 2). The control group (G6) did not develop characteristics lesions of NE. The G7 and G8 given fish meal from day 7 onward along with challenge of ACP and GCP strain respectively, when observed for gross NE lesions have shown 8 out of 10 chicks infected in each group (4 chick: score 3, 4 chicks: score 2). The G9 in which coccidia was given at day 9 along with fishmeal induced non-characteristic lesions of NE in only 2 out of 10 chicks. The G10 and G11 groups included all predisposing factors, i.e., coccidia + fish meal along with type A and type G *C. perfringens* strains. Group 10 (fish meal + Coccidia + ACP) had developed gross lesions in 9 out 10 chicks (3 chicks: score 4, 6 chicks: score 3) while G11 (fish meal + Coccidia + GCP) had developed gross lesions in 10 out 10 chicks (6 chicks: score 4, 4 chicks: score 3). The control group (G12) has not shown typical NE lesions in the chicks at 20th day (Table 5).

**Figure 3:**
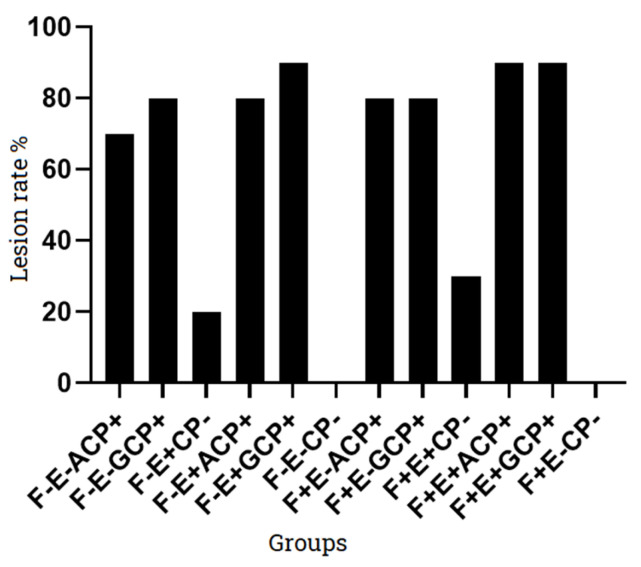
NE lesion rate in disease-induced groups. Note: F: Fish meal; E: *Eimeria* strain; ACP: *C. perfringens* type A; GCP: *C. perfringens* type G; CP: *C. perfringens*; “-”: not added; “+”: added.

**Figure 4:**
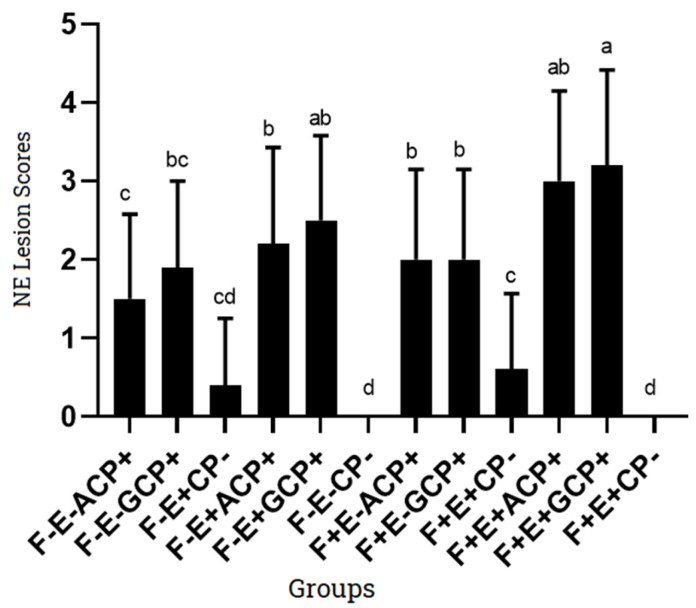
NE lesion score (Mean values) in disease induced groups. Note: F: Fish meal; E: *Eimeria* strain; ACP: *C. perfringens* type A; GCP: *C. perfringens* type G; CP: *C. perfringens*; “-”: not added; “+”: added. Small alphabets indicate significant results in different treatment/control groups.

Three to four days post-challenge with CP (ACP and GCP), the chicks showed decreased activity, depression, reluctance to move, loss of appetite, diarrhea, and ruffled feathers. The chicken had obvious intestinal mucosal congestion and swelling and intestinal mucosal necrotic lesions, and the walls of intestine were thin and full of gas (Figure 3 and Figure 4).

### 3.7. Histopathological Changes in the Small Intestine (Disease Induced Chicks)

**Figure 5:**
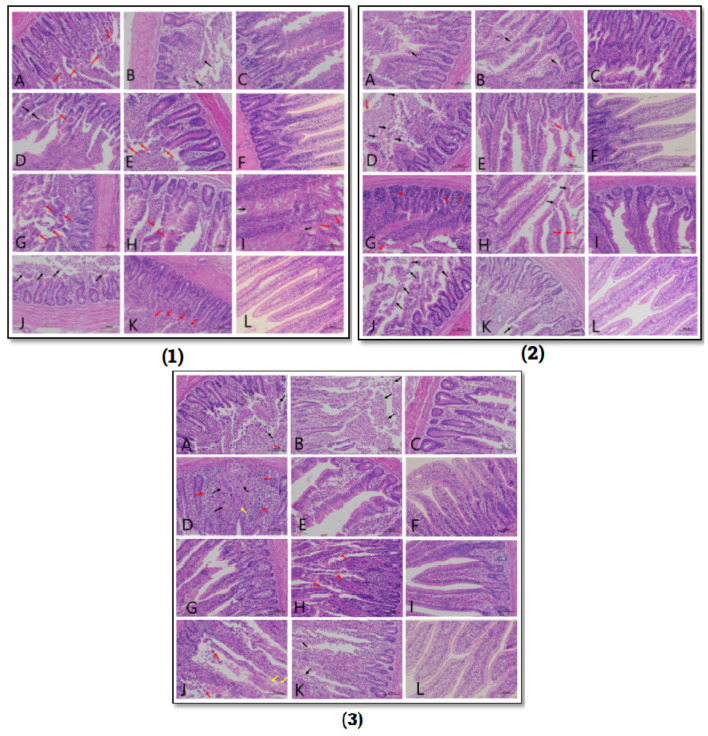
Tissue sections of (**1**): Duodenum (**2**): Jejunum (**3**): Ileum. A1,A2,A3: G1(F-E-ACP+): B1,B2,B3: G2(F-E-GCP+); C1,C2,C3; G3(F-E+CP-); D1,D2,D3: G4(F-E+ACP+); E1,E2,E3: G5(F-E+GCP+); F1,F2,F3: G6(F-E-CP); G1,G2,G3: G7(F+E-ACP+); H1,H2,H3: G8(F+E-GCP+); I1,I2,I3: G9(F+E+CP-); J1,J2,J3: G10(F+E+ACP+); K1,K2,K3: G11(F+E+GCP+); L1,L2,L3: G12(F+E-CP-).

Pathological lesions vary in the disease induction model groups 3 days post challenge. In the ACP group (G1), the mucosal layer of villi was damaged, villi were incomplete, having unclear boundaries, and epithelium was shed. In the GCP group (G2), the lesions were more severe, and the villi and glandular structures were destroyed with incomplete and unclear boundaries. A large number of villous epithelium and lamina propria was shed. The lesions in the coccidia-infected group (G3) were mild having villous epithelium separated from lamina propria at a few areas. The morphology was clear; the epithelial cells were loosely arranged and shed to some extent. The severity of lesions increased in the presence of other predisposing factors. In the coccidia + ACP group (G4), there were more diffuse lymphoid tissues in the mucosal layer, and more villous epithelium was separated from the lamina propria. The coccidia + GCP group (G5) showed a large number of diffuse lymphoid tissues in the mucosal layer, the boundaries of local villi were blurred, the glandular epithelium at the bottom of the mucosal layer was thinned, and the epithelial cells were shed. In the fishmeal + GCP group (G8), the mucosal layer was severely damaged, the villi were incomplete, the villi boundaries were blurred, a large number of villus epithelial cells and lamina propria cells were shed, and there were many diffuse lymphoid tissues in the mucosal layer. A large number of villous epithelial cells fell off, and the gap between the villous epithelium and the lamina propria was widened. In the fishmeal + ACP group (G7), the severity of lesions mentioned above was less. In the fish meal + coccidia + ACP group (G10), a large number of diffuse lymphoid tissues were seen locally, the mucosal layer was severely damaged in a large area, the villi structure was damaged, the boundaries were unclear, and a large number of cells fell off. Intestinal gland expansion is occasionally seen in the mucosal layer, and necrotic cell debris can be seen in the gland cavity. In the fish meal + coccidia + GCP group (G11), a small number of lymph nodes were seen along with diffuse lymphoid tissue, the mucosal layer was seriously damaged, and many villus epithelial cells and lamina propria cells were shed (Figure 5).

### 3.8. Antimicrobial Susceptibility Results for C. perfringens Type G (D25 & MZI) Strains

Two *C. perfringens* type G isolates, i.e., D25 (Anhui province) and MZI (Guangdong province) were used in susceptibility testing. D25 strain was found susceptible to amoxicillin, ampicillin, cefotaxime, ciprofloxacin, enrofloxacin, ofloxacin, and penicillin. Ceftriaxone and cephalexin drugs were found to have intermediate susceptibility while amikacin, doxycycline, florfenicol, gentamicin, kanamycin, metronidazole, neomycin, nystatin, polymyxin B, streptomycin, and tetracycline were resistant against *C. perfringens* D25 strain.

MZI strain was found to have susceptibility against amoxicillin, ampicillin, cefotaxime, ceftriaxone, ciprofloxacin, enrofloxacin, florfenicol, ofloxacin, and penicillin; intermediate susceptibility against cephalexin, gentamicin, and kanamycin; and resistance against amikacin, doxycycline, metronidazole, neomycin, nystatin, polymyxin B, streptomycin, and tetracycline drugs (Table 6).

## 4. Discussion

The isolation rate for *C. perfringens* in this study from the intestinal samples collected during May 2020 to September 2020 was 25.4% (44/173). The samples were collected from commercial farms in Anhui, Guangdong, Guangxi, and Fujian province, China. The isolation rate was found to be almost similar to the published data from Taiwan and Central China (29.6% and 23.1%, respectively) [30,31]. Another study performed in Canada involving two commercial chicken farms also reported *C. perfringens* in 24.7% and 23.3% chicken samples, respectively, which is similar to the above-mentioned results [32]. Data from other regions have reported higher prevalence rate for *C. perfringens* in chicken as well as in other species [25], e.g., studies from Egypt and Jordan reported 57.9% and 43.2% positivity rate for *C. perfringens* isolates [33,34]. In this study, difference in the prevalence rate of *C. perfringens* on different farms was also observed. Farms located in Fujian province have significantly higher prevalence of *C. perfringens* than other locations. These variations are quite common keeping in view different management and hygiene measures and practices adopted on different farms.

The genotyping has characterized the positive isolates into either type A or type G. No isolate was found positive for *beta*, *epsilon*, *iota*, *tpeL,* and *cpe* toxin genes. A total of 95.45% (42 isolates) isolates were type A while the remaining 4.54% (2 isolates) were type G. Xueqin et al. reported 5.3% isolates as type G, while a higher percentage (28%) was detected in another study [35]. Studies from other countries have reported a 70% detection rate for *C. perfringens netB* gene in diseased chicken [17]. There is much difference in the isolation rate of *C. perfringens* types across different regions, i.e., there are variations in the prevalence rates of *C. perfringens* [36]; likewise, type A is the predominant type in many countries including China, Pakistan etc. [29,30,31,37,38]. The high prevalence of *alpha* gene (type A) also signifies the importance of incorporating this toxinotype in vaccine strains along with type G and also suggests the use of an oil-based/montanide adjuvanted vaccine, rather than water-based alum precipitated vaccines, as proven successful in case of other clinically important diseases in Pakistan [39]. The main reason for this might be that type A (*alpha* gene) is chromosomally located while other toxin genes, mainly *beta*, *epsilon*, *iota*, *tpeL*, *netB,* and *enterotoxin* genes, are present on plasmids. Plasmids might be lost during culturing and passaging process, thereby affecting their positive detection as well as higher prevalence rates [40]. Quantitative real-time PCR (qPCR) presents precision as well as higher sensitivity and specificity. It was therefore used in this study to carry out molecular identification and confirmation, as it gives a positive detection rate from low sample content also. Moreover, it enable the researchers to obtain the precise results quickly.

Different studies from various regions of the world have found the *netB* gene in *C. perfringens* isolates from chicken. A study from USA showed that 60% of strains collected from NE cases contained the *netB* gene [41]. Wenjuan et al. collected isolates from different regions of China and isolated 199 strains of *C. perfringens*, of which only six strains carried the *netB* gene. The data indicated that type G strains are more prevalent in studies from other countries as compared to China. This study isolated only two *C. perfringens* type G isolates from NE diseased chicken on commercial farms located at four different places in China. This study proved *C. perfringens* type G as well as type A, as the main toxinotypes that can be used to produce NE lesions. Cooper et al. established a successful animal model using *C. perfringens* type A [19] and Keyburn et al. conducted animal model experiments using wild type *C. perfringens* strain possessing *netB* toxin genes and later on using mutant strains having only *netB* toxin gene and proved the involvement of this gene in causing typical NE lesions [42,43,44]. More researchers insisted on the presence of *netB* gene as a vital factor leading to NE [20]. In another study, a Japanese type G isolate was found to be critical in causing clinical signs of NE disease [45]. It is also interesting to note that type G strains having the *netB* toxin gene have also been isolated from healthy chicken, as reported in many studies across the world [41,46]. The researchers therefore put forward other significant factors commonly called predisposing factors, which might be very critical in inducing as well as enhancing the severity of NE in many cases [11,12]. These factors were found critically important and were observed in both natural as well as experimental disease induction models. The leading co-factors include infection with coccidia, diet source, protein content, as well as to some extent other hygiene compromising strategies/factors [13].

The experimental induction of NE has been successful with and without predisposing factors using *C. perfringens* type G strain (7.5 × 10^8^ cfu/mL) along with coccidia (0.5 × 10^4^ oocysts/mL) and fish meal (1:1). Coccidia in this study were found to induce intestinal damage, epithelial layer destruction, leakage of plasma proteins, mucin secretion, along with gas production. Coccidia infection on day 9 followed by challenge with *C. perfringens* strains increase the frequency and severity of lesions. Many researchers have used a co-infection strategy and found that Eimeria spp. and *C. perfringens* both promoted the occurrence of NE [47,48]. A high-protein diet somehow changes the gut nutritional environment and intestinal microflora, thereby favors the proliferation of *C. perfringens*, which induces necrotic enteritis lesions in chicken. The predisposing factors increased the severity of lesions and gave significant results. Results have shown significant difference in the NE lesion score of G11 when compared with G1, G2, G4, G5, G7, G9, and G10 (*p* < 0.05). Compared to the control group (G6), the CP challenge group (G1 and G2) had significant difference (*p* < 0.05). In groups challenged with CP alone, there was no significant difference between groups challenged with type A (G1) or type G (G2) (*p* > 0.05). However, there was a difference in severity of the lesions, i.e., the type G strain produced more severe NE lesions as compared to type A. For type A strain, the lesions induced in the presence of coccidia (G4) and fish meal (G7) were more severe and had significant difference as compared to the group (G1), in which ACP alone was used for challenge. It means that fish meal played a significant role in the occurrence of NE in the absence of coccidia infection. The association between fishmeal and NE could be higher zinc concentration, which consequently increases production of alpha toxins. It is also known that high levels of dietary fishmeal contribute to the growth of *C. perfringens,* increasing its number in the caecum and ileum of birds, thereby suggesting its role as a predisposing factor [49]. The challenge with coccidia in the absence of fish meal also increased the severity of NE (*p* < 0.05). Similarly, the type G strain induced more severe lesions in the presence of predisposing factors, i.e., fishmeal and coccidia. In this study, 3–4 days post challenge with *C. perfringens*, chicks were found depressed, fatigued, having ruffled feathers with subsequent diarrhea [50]. Gross lesions were seen frequently in jejunum and ileum. The mucosal layer of intestine was damaged, villi were incomplete, with unclear boundaries and shedding of villous epithelial cells, and lamina propria was seen [49].

*C. perfringens* type G strains identified in this study were found susceptible to amoxicillin, ampicillin, cefotaxime, ciprofloxacin, enrofloxacin, ofloxacin, and penicillin. Amoxicillin, ampicillin, cefotaxime, ofloxacin, and penicillin were found effective against *C. perfringens* isolates in many other studies also [22,37,51,52]. Wei et al. observed increased antimicrobial resistance to tetracycline, florfenicol, gentamycin, and enrofloxacin [22]. Our results also indicated multidrug resistance profile, comprising resistance to amikacin, doxycycline, metronidazole, neomycin, nystatin, polymyxin B, streptomycin, and tetracycline in both type G strains. Antimicrobial activity testing of 230 *C. perfringens* isolates reported susceptibility to amoxicillin, ampicillin, cefepime, and norfloxacin in 100, 91, 89, and 81% isolates, respectively, while resistance was observed in case of streptomycin in 98% isolates [51]. Variable sensitivity patterns were also seen in studies revealing all *C. perfringens* isolates sensitive to gentamicin [52] and neomycin [53] in contrast to our findings, while the same pattern of sensitivity was observed in isolates for penicillin [54] and fluoroquinolones [55,56,57]. Antimicrobials including amoxicillin, enrofloxacin, and florfenicol were found to have lower MIC_50_ values, i.e., 0.125, 0.25, and 2 lg/mL and were found effective in similar studies (2). Studies conducted to find the most effective antimicrobial drug against *C. perfringens* in poultry reported amoxicillin as the best choice in China, Jordan, Sweden, and USA [34,58,59,60].

## 5. Conclusions

This study provides the ideal model to study the pathogenesis of NE in which a clinically significant number of challenged chicks (90–100%) developed characteristic signs as well as gross lesions of NE without mortality. The multiple doses of *C. perfringens* enriched using standardized protocol induces NE in a significant proportion (60%) of chicks. It can be inferred from this animal model that NE can be induced in SPF chicks without the use of other predisposing factors. Multiple-drug resistance was observed in both *C. perfringens* type G strains in addition to having variable susceptibility against some antimicrobial agents. It is further observed that a better understanding of the presence of toxin genes and antimicrobial resistance is needed to carefully control increasing resistance issues in pathogenic clostridia.

## Figures and Tables

**Table 1:** Quantitative real-time PCR (qPCR) primer sequence for *C. perfringens* toxinotyping.

Toxin Genes	Primers	Primers Sequence (5′–3′)	Genbank Acc. Number	Fragment Size (bp)
CPA	CPA F	GATGGAAAAATTGATGGAAC	L43545	136
	CPA R	CATGCATGTTCTCTTTTAAAAT		
CPB	CPB F	TATTCCTAAAAATACAATTTCTCA	KP064410	212
	CPB R	CTGTAAATTTTGTATCCCATGA		
ITX	ITX F	CTAGCCCAAATTCTATATTTTTGTA	X73562	222
	ITX R	GTTGGTAAAAGATGTGTTTTAATAG		
ETX	ETX F	TTAGTTTATCGGATACAGTAAAT	M95206	242
	ETX R	ATAATCTTATTTTATTCCTGGTG		
TPEL	TPEL F	GCGATTATGAAACTATTATATGGTA	EU848493	168
	TPEL R	TAACTTCCATTCTTTCTCTATA		
CPE	CPE F	GATAGCTTAGGAAATATTGATCAAG	X81849	217
	CPE R	GTAAATTAAGCTTTTGAGTCCA		
NetB	NetB F	TGAGACTAAGGACGGTTATAATA	FJ189497	214
	NetB R	TTGATATTCAACTATTATTACAGAT		

**Table 2:** Trial design (animal model studies).

Group/s	No. of Chicks	Group Details	Dietary Factor Added (Day/s)	Coccidia Challenge Day/s	*C. perfringens* Challenge Day/s	Sampling Time (Day)
1	10	F-E-ACP+	-	-	14, 15, 16, 17	20
2	10	F-E-GCP+				
3	10	F-E+CP-		9	-	
4	10	F-E+ACP+			14, 15, 16, 17	
5	10	F-E+GCP+				
6	10	F-E-CP-		-	-	
7	10	F+E-ACP+	8–20		14, 15, 16, 17	
8	10	F+E-GCP+				
9	10	F+E+CP-		9	-	
10	10	F+E+ACP+			14, 15, 16, 17	
11	10	F+E+GCP+				
12	10	F+E-CP-		-	-	

Note: F: Fish meal; E: *Eimeria necatrix* strain; ACP *C. perfringens* type A strain; GCP: *C. perfringens* type G; CP: *C. perfringens*; “-”: not added; “+”: added.

**Table 3:** Antibiotic susceptibility standards.

S.No	Inhibition Zone Diameter	Sensitivity
1.	<10 mm	Resistant (R)
2.	10~15 mm	Moderately sensitive (I)
3.	>15 mm	Highly Sensitive (S)

**Table 4:** Isolation results of *Clostridium perfringens* from diseased chicken.

Sr. No	Samples (Location)	No. of Samples Collected	Samples Positive for *C. perfringens*	Positivity Rate for *C. perfringens* (%)
1.	Anhui	50	10	20%
2.	Guangdong	56	11	19.6%
3.	Fujian	19	12	63.1%
4.	Guangxi	48	11	22.9%
	Total	173	44	25.4%

**Table 5:** NE lesion scores (Mean ± SD) in chicks challenged with *C. perfringens* strains with and without predisposing factors.

Groups	Chicks (No)	Lesion Score (No of Chicks)							Mean ± Standard Deviation
		0+	1+	2+	3+	4+	5+	6+	
G1(F-E-ACP+)	10	3	2	5	0	0	0	0	1.5 ± 1.08
G2(F-E-GCP+)	10	2	1	7	0	0	0	0	1.9 ± 1.10
G3(F-E+CP-)	10	8	0	2	0	0	0	0	0.4 ± 0.85
G4(F-E+ACP+)	10	2	0	2	6	0	0	0	2.2 ± 1.23
G5(F-E+GCP+)	10	1	0	4	4	1	0	0	2.5 ± 1.08
G6(F-E-CP-)	10	7	3	0	0	0	0	0	0.0 ± 0.0
G7(F+E-ACP+)	10	2	0	4	4	0	0	0	2.0 ± 1.15
G8(F+E-GCP+)	10	2	0	4	4	0	0	0	2.0 ± 1.15
G9(F+E+CP-)	10	0	0	2	0	0	0	0	0.6 ± 0.97
G10(F+E+ACP+)	10	1	0	0	6	3	0	0	3.0 ± 1.15
G11(F+E+GCP+)	10	0	0	0	4	6	0	0	3.2 ± 1.22
G12(F+E-CP-)	10	6	4	0	0	0	0	0	0.0 ± 0.0

Note: F: Fish meal; E: *Eimeria necatrix* strain; ACP: *C. perfringens* type A strain; GCP: *C. perfringens* type G; CP: *C. perfringens*; “-”: not added; “+”: added.

**Table 6:** Antibiotic susceptibility testing of *C. perfringens* type G strains (D25 and MZI) against various drugs.

*C. perfringens*	Strain D25		Strain MZ1	
Antibiotics	Zone of Inhibition (mm)	Sensitivity	Zone of Inhibition (mm)	Sensitivity
Kanamycin	0	R	11	I
Polymyxin B	0	R	0	R
Ceftriaxone	15	I	16	S
Amoxicillin	26	S	34	S
Tetracycline	0	R	0	R
Amikacin	0	R	0	R
Neomycin	0	R	0	R
Penicillin	24	S	28	S
Florfenicol	0	R	16	S
Enrofloxacin	16	S	19	S
Streptomycin	0	R	0	R
Ampicillin	25	S	26	S
Metronidazole	0	R	0	R
Ciprofloxacin	20	S	20	S
Gentamicin	0	R	11	I
Cefotaxime	22	S	20	S
Nystatin	0	R	0	R
Ofloxacin	22	S	20	S
Doxycycline	0	R	0	R
Cephalexin	11	I	11	I

## Data Availability

Most of the data generated during this study are included in this article. The rest can be provided by the corresponding author upon request.
